# Photo-crosslinking of doped magic-sized nanoclusters for the construction of enhanced electrochemiluminescence biosensors†

**DOI:** 10.1039/d4sc07800d

**Published:** 2025-01-22

**Authors:** Junjun Ge, Tengyue Yin, Haoyang Zhang, Yue Cao, Juan Liu, Jun-Jie Zhu, Yang Zhou, Yuanyuan Wang

**Affiliations:** a State Key Laboratory of Coordination Chemistry, School of Chemistry and Chemical Engineering, Nanjing University Nanjing 210093 China wangyy@nju.edu.cn; b Key Laboratory for Organic Electronics & Information Displays, Institute of Advanced Materials, Nanjing University of Posts & Telecommunications (NJUPT) Nanjing 210023 PR China iamyangzhou@njupt.edu.cn; c State Key Laboratory of Pollution Control and Resource Reuse, School of the Environment, Nanjing University Nanjing 210023 China; d School of Environmental & Chemical Engineering, Jiangsu University of Science and Technology Zhenjiang 212100 China

## Abstract

Semiconductor magic-sized nanoclusters (MSCs) possess atomic-level compositional precision and ultrasmall dimensions, allowing accurate modulation of electrochemiluminescence (ECL) properties, essential for advanced bioanalytical applications. However, low intrinsic ECL intensity and poor stability in bipolar electrode (BPE)-ECL systems hinder their broader use. In this work, we addressed these limitations through doping and direct optical crosslinking strategies, achieving a 24-fold boost in the ECL signal and a fivefold stability increase for doped (CdS)_34_:Ag MSCs compared with original (CdS)_34_ MSCs. The resulting BPE-ECL biosensing platform was used for the sensitive detection of glucose with a linear detection range of 10 μM to 1 mM and a detection limit of 3.64 μM. This approach provides a robust strategy to enhance MSC-based ECL biosensing, paving the way for ultrasensitive, stable biosensors for clinical diagnostics and bioanalysis.

## Abstract 2

We developed a bipolar electrode-based ECL sensing platform using doping and direct optical crosslinking of semiconductor magic-sized nanoclusters, achieving significant improvements in both ECL intensity and stability.

## Graphical abstract

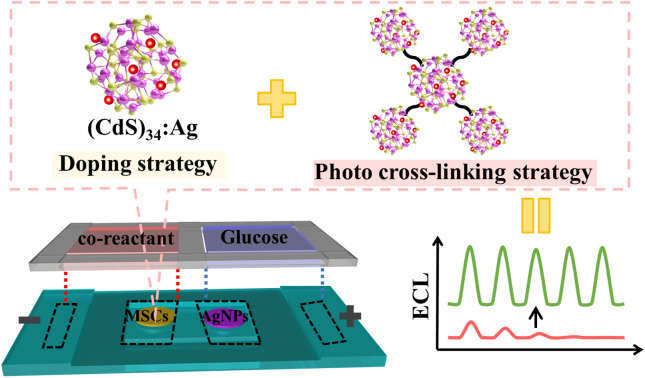

## Introduction

Electrochemiluminescence (ECL), known as electrogenerated chemiluminescence, has emerged as a powerful analytical technique with broad applications.^1–5^ Among various ECL approaches, bipolar electrode-based ECL (BPE-ECL) stands out due to its unique two-electrode configuration, where electrodes are spatially separated, offering several distinct advantages over the traditional three-electrode system.^6–8^ Specifically, BPE-ECL significantly minimizes electron transfer barriers between aqueous and organic phases, thereby enhancing charge transfer efficiency.^9,10^ In addition, its modular structure facilitates the integration of multiple analytical techniques within distinct electrode regions, enabling the design of sophisticated array-based biosensors.^11–13^ This versatility has generated significant interest in BPE-ECL, establishing it as a promising approach for advanced biosensing applications.

In the BPE-ECL system, the emitter plays a pivotal role, with its careful selection and design being essential to achieving optimal ECL performance. Magic-sized nanoclusters (MSCs), which are ultrasmall, monodisperse nanomaterials with countable atoms, exhibit discrete growth characteristics and unique, size-dependent optical properties.^14–16^ Given their small size (<2 nm) and tunable optical properties, MSCs are considered ideal emitters for ECL detection.^17,18^ In our previous work, we established that the ECL intensity in (CdS)_34_ and (CdSe)_34_ MSCs could be modulated by surface interface modifications.^19^ However, despite these enhancements, the ECL intensity of these MSCs remains comparatively low, limiting their sensitivity in application. Furthermore, the inherent instability of MSCs on the electrode surface, where they tend to detach during detection, poses practical challenges. Thus, addressing both the signal intensity and the film stability of MSCs remains essential for advancing their use in high-sensitivity ECL detection.

Doping technology offers a promising approach to enhancing the ECL emission through several mechanisms. Key among these are the improvement of emitter conductivity, fine-tuning of energy level alignment, and increased efficiency of reactions between the emitter and co-reactants.^20–22^ In addition, doping can reinforce emitter stability and reduce quenching effects, collectively boosting the ECL signal intensity. However, achieving effective doping while maintaining the structural stability of MSCs remains a major challenge in this field.^23^ Photo-crosslinking alternatively provides a viable means to enhance the stability and adhesion of materials to their substrates, critical factors for reliable ECL performance.^24,25^ This method strengthens interactions between the material and substrate, improving mechanical stability.^26^ Yet, MSCs, due to their extremely small size and high surface energy, are particularly sensitive to environmental changes. Conventional photosensitive additives, such as photoacid generators (PAGs), can potentially damage MSCs. While widely used in nanocrystal patterning, applications of photo-crosslinking in MSC-based systems are still relatively unexplored, indicating a need for further investigation to adapt these techniques in cluster-based systems.^25^

**Scheme 1:**
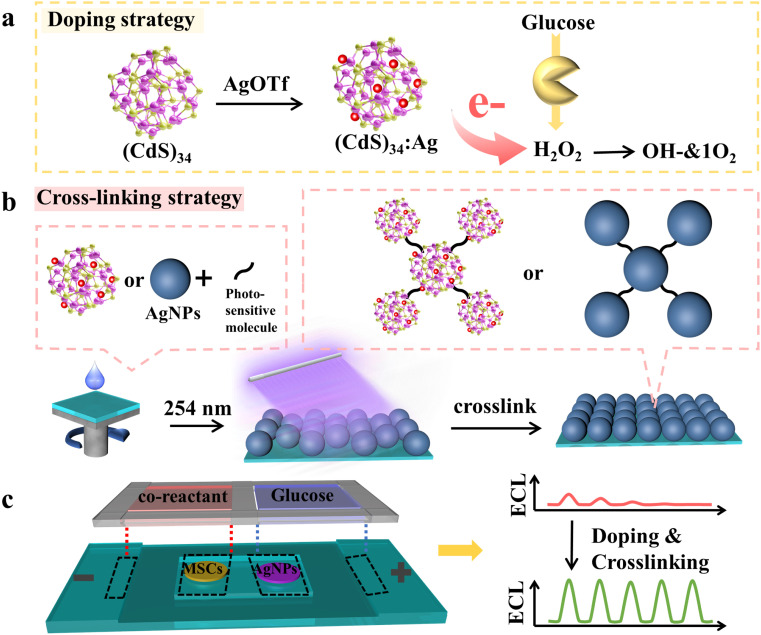
Schematic illustrations of (a) the doping strategy of (CdS)_34_ MSCs and the detection principle of glucose. (b) Cross-linking strategy of (CdS)_34_:Ag MSCs and Ag NPs. (c) Construction of the BPE system.

In this work, we introduced a crosslinked doped-MSC strategy to construct an advanced BPE-ECL sensing platform, significantly enhancing ECL performance for glucose detection (Scheme 1). Specifically, silver-doped (CdS)_34_ MSCs ((CdS)_34_:Ag MSCs) were synthesized by incubating (CdS)_34_ MSCs with silver trifluoromethanesulfonate (Ag(OTf)). This doping approach amplified the ECL intensity of (CdS)_34_ MSCs by 24-fold compared to their undoped counterparts, making the MSCs potential emitters for ECL applications (Scheme 1a). To improve emitter stability, we introduced photosensitive molecules to crosslink MSCs, which anchored them securely onto the electrode surface. In addition, crosslinked Ag NPs served as catalysts, further stabilizing the BPE-ECL devices (Scheme 1b). Using these strategies, we constructed a robust BPE-ECL system with (CdS)_34_:Ag MSCs as primary emitters for highly sensitive glucose detection (Scheme 1c). The platform achieved an ECL signal intensity of 8000 a.u., a 20-fold increase over traditional MSC-based ECL systems. The ECL demonstrated excellent stability over five consecutive scans. With a broad linear detection range from 10 μM to 1 mM and a low detection limit of 3.64 μM for glucose, this BPE-ECL biosensor showcased outstanding analytical performance. Our doping and crosslinking strategy effectively addresses the limitations of traditional MSC-based ECL, expanding MSC functionality and advancing the design of high-performance ECL biosensors. This approach opens new avenues for future biosensor development and innovation.

## Results and discussion

The MSCs studied in this work were synthesized using established protocols, involving reactions of a cadmium precursor (CdCl_2_) dissolved in octylamine (OTA) with sulfur dissolved in OTA.^19,27^ The resulting products exhibited a UV-vis absorption spectrum with two sharp peaks at 365 and 335 nm (Fig. S1a†), consistent with the typical absorption of (CdS)_34_ MSCs.^28^ To confirm the chemical composition, inductively coupled plasma optical emission spectroscopy (ICP-OES) analysis revealed a Cd : S molar ratio of 1 : 1, supporting the (CdS)_*x*_ stoichiometry (Table S1†). In addition, laser desorption ionization mass spectrometry (LDI-MS) identified the number of cluster atoms as *x* = 34, as shown in Fig. S1b.†

### Preparation and characterization of Ag doped (CdS)_34_ MSCs

The synergistic effect of bimetallic components significantly enhances the ECL performance, as the combination of cadmium (Cd) and doped silver (Ag) improves charge transfer efficiency and increases the number of active sites, resulting in higher ECL intensity.^29–31^ However, despite the potential benefits, controlled doping of MSCs remains challenging due to their structural instability and atomically precise small size. In this study, a mild post-synthesis doping strategy was employed. Initially, (CdS)_34_ MSCs were synthesized, followed by incubation with Ag(OTf) for 3 hours. After purification, the (CdS)_34_:Ag MSCs were successfully obtained.

**Fig. 1:**
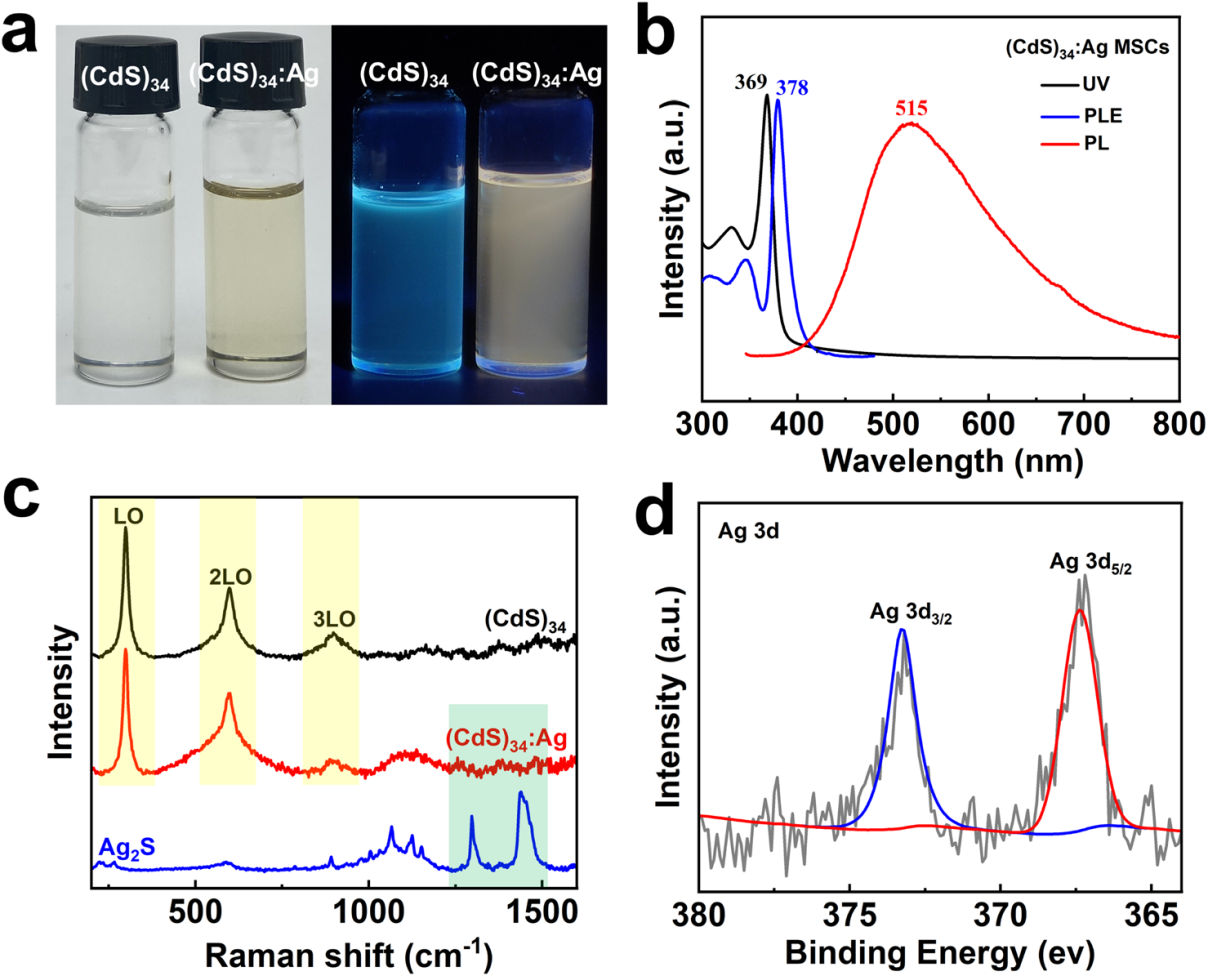
(a) Photographs of (CdS)_34_ and (CdS)_34_:Ag MSCs under natural and UV light. (b) UV-vis absorption, PL and PLE spectra of (CdS)_34_:Ag MSCs. (c) Raman spectra of (CdS)_34_, (CdS)_34_:Ag MSCs and Ag_2_S. (d) High-resolution X-ray photoelectron spectra of (CdS)_34_:Ag MSCs in the Ag 3d electron binding energy range.

To confirm the successful doping, several experiments were conducted. The fluorescence photographs showed a color change from blue to green under UV light upon doping (Fig. 1a). Correspondingly, the photoluminescence (PL) emission peak of (CdS)_34_ MSCs at 515 nm red-shifted by 35 nm compared to the undoped MSCs (480 nm), with a 13-fold increase in the photoluminescence quantum yield (PLQY). This enhancement and redshift were attributed to the introduction of Ag ions, which affected electron localization and altered the energy level structure of (CdS)_34_ MSCs.^32,33^ Meanwhile, the UV-visible absorption spectrum of (CdS)_34_:Ag MSCs exhibited a 6 nm redshift relative to the undoped MSCs (Fig. 1b), indicating an increased effective radius due to Ag atom incorporation.^23^ As the Ag feeding ratio increased, both UV-visible and fluorescence spectra exhibited a gradual redshift, with the PLQY reaching a maximum at a (CdS)_34_ : Ag(OTf) ratio of 1 : 3 (Fig. S2†).

ICP-OES was employed to determine the doping level of silver in (CdS)_34_ MSCs. Table S1† shows that 12–13.8% silver was successfully incorporated into the (CdS)_34_ MSCs. To further investigate the bonding interactions of silver within the MSCs, Raman spectroscopy was conducted. As shown in Fig. 1c, both undoped (CdS)_34_ MSCs and Ag-doped (CdS)_34_ MSCs exhibited three prominent peaks at approximately 298 cm^−1^ (1LO), 598 cm^−1^ (2LO), and 899 cm^−1^ (3LO). These peaks correspond to longitudinal optical (LO) phonon modes characteristic of Cd–S bonding, and their presence in both doped and undoped samples suggests that silver doping does not disrupt the intrinsic Cd–S bonding structure of the MSCs. Furthermore, no peaks associated with Ag_2_S (typically observed at 1297 and 1435 cm^−1^) were detected in the Raman spectrum of (CdS)_34_ MSCs. This absence confirms that silver does not form Ag–S bonds within the MSC matrix. These findings imply that Ag is incorporated into the (CdS)_34_ MSCs through doping rather than through the formation of Ag_2_S. X-ray Photoelectron Spectroscopy (XPS) determined the forms of the elements present in the (CdS)_34_:Ag MSCs. The Ag 3d electron binding energy spectrum (Fig. 1d) exhibits a doublet at 367.4/373.2 eV, characteristic of Ag(i), indicating that silver does not form Ag nanoclusters or nanocrystals but instead exists as ionic species.^34,35^ Additionally, the XPS spectra of Cd 3d and S 2p confirm that Cd and S in the (CdS)_34_:Ag MSCs samples are present as Cd^2+^ and S^2−^, respectively (Fig. S4†). This suggests that Ag^+^ ions are likely doped into the (CdS)_34_ MSCs through electrostatic interactions.

Verifying the integrity of the host nanomaterials following doping is essential for evaluating the reliability and functionality of the modified materials. ICP-OES results (Table S1†) demonstrated that the ratio of Cd : S in the doped (CdS)_34_ MSCs remained unchanged, consistent with the stoichiometry of the original (CdS)_34_-OTA MSCs. This result confirms that the doping process preserved the overall composition of the MSCs. Furthermore, the photoluminescence excitation (PLE) and UV-visible absorption spectra of the doped MSCs exhibited similar peak shapes to those of the undoped MSCs. This observation indicates that the fundamental bandgap structure of the (CdS)_34_ MSCs remained intact following doping. The slight red-shift observed between the PLE and absorption spectra was attributed to the contribution of different fine structure states to each signal. This behavior aligns with previously reported findings for other MSCs, such as (CdSe)_13_ (Fig. 1b).^23^

The incorporation of heteroatoms into nanoclusters often leads to changes in their structure and morphology. However, our MSCs proved to be an exception.^36^ The XRD patterns of (CdS)_34_ and (CdS)_34_:Ag MSCs, as shown in Fig. S3c,† exhibited only minimal variations, strongly indicating that the structural integrity of (CdS)_34_ MSCs remained unchanged despite the doping process. Transmission electron microscopy (TEM) images confirmed that the morphology of (CdS)_34_ MSCs was preserved, with no observable changes in shape or size (approximately 3 nm) following the introduction of Ag (Fig. S3a and b†). These combined findings provide strong evidence for successful silver doping in (CdS)_34_ MSCs while maintaining their structural and elemental stability.

### Enhancement of the ECL intensity of (CdS)_34_ by doping

**Fig. 2:**
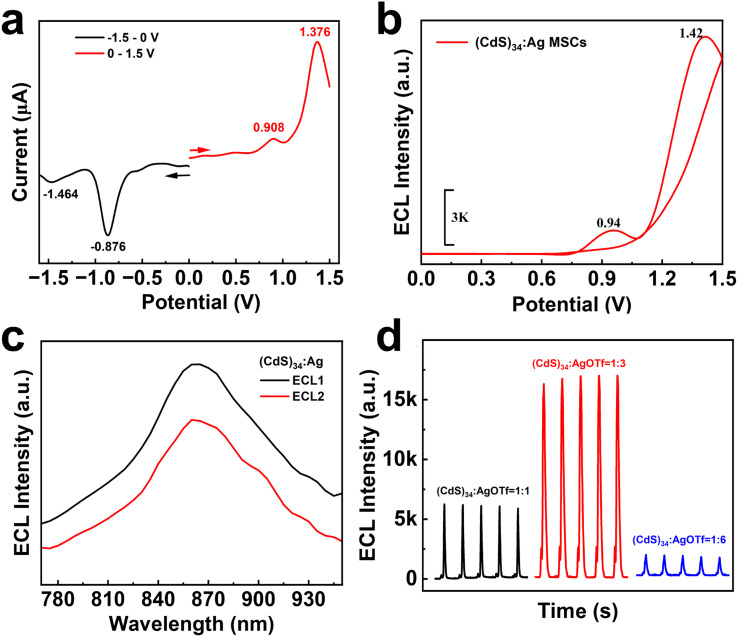
(a) DPV curve of (CdS)_34_:Ag MSCs, (b) ECL-E curve of (CdS)_34_:Ag MSCs. (c) ECL spectra of (CdS)_34_:Ag MSCs at different potentials (ECL1 at 0.9 V; ECL2 at 1.4 V). (d) ECL intensity of (CdS)_34_:Ag MSCs with different feeding ratios ((CdS)_34_ : AgOTf = 1 : 1, 1 : 3 and 1 : 6).

To elucidate the ECL mechanism of (CdS)_34_:Ag MSCs, a detailed electrochemical analysis was conducted. As illustrated in Fig. 2a, the differential pulse voltammogram (DPV) measurements of (CdS)_34_:Ag MSCs revealed two distinct, irreversible oxidation peaks at 0.908 V and 1.376 V, designated as O1 and O2, respectively. These peaks were attributed to the oxidation reactions of the components in the (CdS)_34_:Ag MSCs. By drawing parallels to the interpretation of DPV profiles in (CdS)_34_ MSCs, as previously reported, O1 and O2 were assigned to the redox transitions of the (CdS)_34_:Ag^+^/(CdS)_34_:Ag^0^ and (CdS)_34_:Ag^2+^/(CdS)_34_:Ag^+^ oxidation couples, respectively (eqn (3) and (6)). Notably, the corresponding O1 and O2 exhibited close agreement with the observed ECL emission potentials, designated as ECL1 (0.94 V) and ECL2 (1.42 V) in Fig. 2b. This alignment strongly suggested that ECL1 and ECL2 arose from the excited states of (CdS)_34_:Ag^+^ and (CdS)_34_:Ag^2+^, respectively, thus providing crucial insights into the luminescence behavior of (CdS)_34_:Ag MSCs under electrochemical excitation. It is noteworthy that the co-reactant TPA underwent an oxidation reaction, generating a TPA radical cation (TPA˙^+^), which rapidly deprotonated to generate the highly reducing species TPA˙ (eqn (1) and (2)). In the ECL1 pathway for (CdS)_34_:Ag MSCs, the surface atoms of (CdS)_34_:Ag^+^ interacted with TPA˙, accepting an electron to yield the excited state (CdS)_34_:Ag*, which subsequently relaxed to its ground state while emitting light at 860 nm (eqn (3)–(5)), as depicted in Fig. 2c. In contrast, the ECL2 mechanism for (CdS)_34_:Ag MSCs was more intricate. The (CdS)_34_:Ag^2+^ surface atoms could accept a single electron from TPA˙, resulting in the formation of (CdS)_34_:Ag^+^*, or alternatively, they may accept two electrons directly to yield (CdS)_34_:Ag*. Both ECL1 and ECL2 displayed identical emission wavelengths (860 nm, as shown in Fig. 2c), strongly suggesting that the emissions originate from the same excited state. Consequently, ECL2 was attributed to the relaxation of (CdS)_34_:Ag* back to its ground state, following a two-electron transfer process (eqn (6)–(9)). The specific process is illustrated as follows:

1 TPA-e^−^ → TPA˙^+^

2 TPA^+^˙ → TPA˙ + H^+^

3 (CdS)_34_:Ag-e^−^ → (CdS)_34_:Ag^+^

4 (CdS)_34_:Ag^+^ + TPA˙ → (CdS)_34_:Ag*

5 (CdS)_34_:Ag* → (CdS)_34_:Ag + *hv*

6 (CdS)_34_:Ag^+^-e^−^ → (CdS)_34_:Ag^2+^

7 (CdS)_34_:Ag^2+^ + TPA˙ → (CdS)_34_:Ag^+^

8 (CdS)_34_:Ag^+^ + TPA˙ → (CdS)_34_:Ag*

9 (CdS)_34_:Ag* → (CdS)_34_:Ag + *hv*

Importantly, the ECL intensity of (CdS)_34_:Ag MSCs enhanced significantly with a higher level of Ag doping, reaching a maximum at a (CdS)_34_ to AgOTf feed ratio of 1 : 3. At this point, the ECL2 value peaked at 16 800 a.u., representing a 24-fold enhancement compared to that of the (CdS)_34_-OTA sample (ECL2 of 700 a.u.). This improvement was attributed to the synergistic effect of the bimetallic system (Fig. 2d and S5†). Therefore, based on these findings, we selected the (CdS)_34_ MSCs prepared at this doping ratio as the luminophore for subsequent detection experiments. However, the high resistance of the BPE system posed a challenge, as the (CdS)_34_ MSCs exhibited a luminescence potential of up to 10 V, exceeding the available potential window of conventional ECL measurements. To address this limitation, we introduced Ag NPs as catalysts to promote electron transfer and reduce the ECL potential of MSCs. The catalytic mechanism was proposed based on the higher affinity of Ag NPs for H_2_O_2_, which favored the generation of OH- and ^1^O_2_ to further facilitate electron transfer processes. This catalytic effect not only lowered the luminescence potential but also further increased the ECL intensity of the (CdS)_34_:Ag MSCs, enabling improved performance in the subsequent experiments (Fig. S6†).

### Improved ECL stability by a photo-crosslinking strategy

In BPE-ECL devices, a critical challenge lies in improving the adhesion between luminescent materials and between the emitting layer and the indium tin oxide (ITO) electrode surface.^37,38^ Poor adhesion often results in the detachment of the luminescent materials from the electrode surface, significantly compromising ECL performance and long-term stability. Previous approaches to addressing this issue included covalent bonding, grafting and device encapsulation.^39–41^ However, these approaches come with drawbacks since covalent bonding alters the inherent electronic properties of MSC emitters, potentially diminishing their performance, while the introduction of an encapsulation layer can hinder charge transport and complicate the device fabrication. To overcome these limitations, we implemented a photo-crosslinking strategy. This method enhances the mechanical stability of the luminescent layer without affecting the inherent properties of MSC emitters, providing a robust and efficient solution to this long-standing problem.

Direct optical lithography is a photo-patterning technique that does not rely on polymer photoresists.^24,25^ By introducing photosensitive molecules and utilizing radical polymerization, it enables the crosslinking of MSCs. This photoresist-free and amplifier-free crosslinking strategy could minimize the potential damage, preserving the structural and functional integrity of MSCs.^42^ We investigated the impact of crosslinking on the stability of MSC films. The (CdS)_34_:Ag MSCs were mixed with a photosensitive molecule and spin-coated on ITO. Then, the crosslinked samples were exposed to UV light, while a non-crosslinked sample was not. Following this, all films were immersed in toluene and dried. The stability of the films was assessed by examining the presence of residual nanomaterials on the surface under different conditions.

**Fig. 3:**
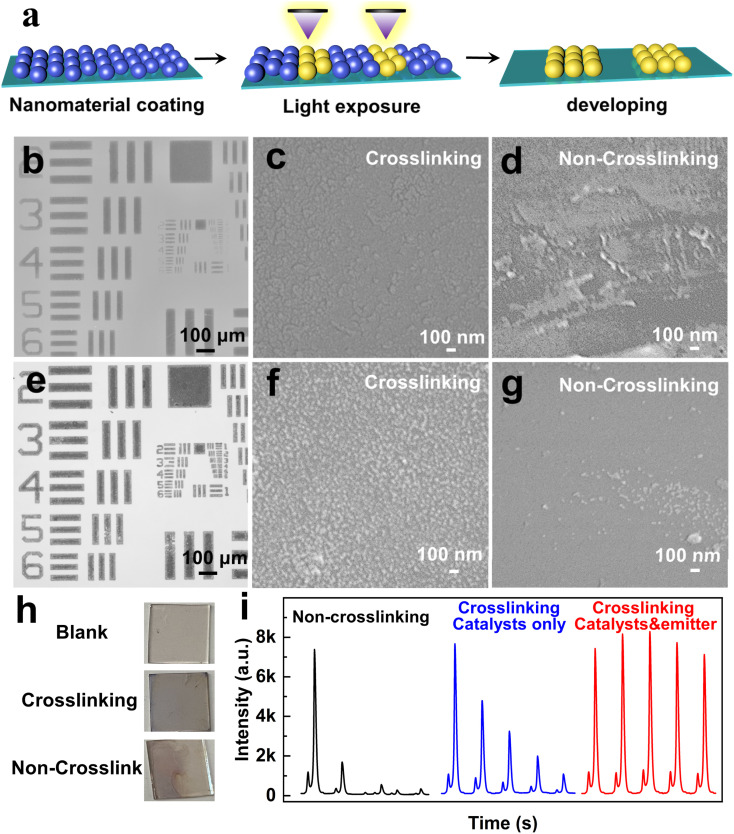
(a) Outline of the processing steps for lithography by crosslinking. Microscopic imaging of lithography by crosslinking for (b) (CdS)_34_:Ag MSCs and (e) Ag NPs. SEM image of (c) (CdS)_34_:Ag MSCs with and (d) without crosslinking; (f) Ag NPs with and (g) without crosslinking; (h) photo of Ag NPs attached to ITO and (i) ECL intensities of the BPE system with different crosslinking conditions.

We utilized a mixture of the MSCs and photosensitive molecule as a photosensitive ink for optical lithography. Under deep UV (254 nm) irradiation, photogenerated carbene radicals initiated nonspecific C–H insertion with the native ligands on MSCs, facilitating crosslinking between adjacent MSCs.^26^ This process rendered the MSCs in the exposed regions insoluble in nonpolar solvents, while the unexposed areas remained soluble and could be washed away with toluene to form precise patterns (Fig. 3a).

The patterning quality is demonstrated in Fig. 3b, where a 100-micron stripe pattern exhibited high fidelity with sharp and clean edges. It is worth noting that the patterning of semiconductor MSCs is considered highly challenging. This difficulty arises from the extremely small size and high surface energy of the MSCs, rendering them highly susceptible to external factors and resulting in pronounced instability. Conventional lithography processes, such as exposure, development, and etching, demand materials with high stability, making this achievement noteworthy. In this approach, UV-triggered radicals generated by the photosensitive molecule effectively couple the MSCs through their native ligands, enabling nondestructive and gentle patterning.^26^ Similarly, Ag NPs were successfully patterned using the same technique, as shown in Fig. 3e. The resulting 100-micron stripe patterns demonstrate high contrast, sharp edges, and excellent fidelity, underscoring the versatility of the photosensitive molecule as a universal crosslinking agent for nanomaterial patterning.

Fig. 3 and S7† reveal that the crosslinked MSCs remained intact at the micron scale after rinsing, showing no signs of breakage or detachment (Fig. S7a†). Zoomed-in images further confirmed a dense and well-defined film structure, indicating excellent stability of the crosslinked films (Fig. 3c). Energy dispersive X-ray spectroscopy (EDS) mapping shows uniformly distributed elements. Meanwhile, the Ag element is much less abundant than both Cd and S elements (Fig. S8†). In contrast, the non-crosslinked (CdS)_34_:Ag MSC film showed a significant material loss after immersion in the electrode detection solution, highlighting its instability without crosslinking (Fig. 3d and S7b†). The EDS mapping reveals a uniform distribution of elements exclusively on the residual film, while the detached portion exhibits no detectable elemental attachment (Fig. S9†). A similar trend was observed for the Ag NP film. The crosslinked Ag NP films remained intact, with no signs of breakage or detachment, whereas the non-crosslinked film exhibited substantial detachment (Fig. 3f, g, S7c and d†). In addition, images of Ag NPs on the ITO surface after immersion revealed that the crosslinked Ag NPs were uniformly and stably distributed across the entire surface, with no noticeable detachment. In contrast, the non-crosslinked Ag NPs dissolved and detached. The above experiments were sufficient to demonstrate that the crosslinking strategy significantly improved the stability of MSCs and Ag NPs on ITO.

One of the most critical advantages of stable nanomaterial film formation is its ability to ensure consistent and reliable ECL signals. As both the emitter (CdS)_34_:Ag MSCs and catalyst Ag NPs are firmly attached to the electrode surface, the ECL intensity decays more slowly. Fig. 3i shows the ECL intensities of the BPE system under different crosslinking conditions. When neither (CdS)_34_:Ag MSCs nor Ag NPs are crosslinked, the ECL intensity dropped sharply by the second scan cycle. Crosslinking only the Ag NPs helps moderate the rate of decay, but a gradual decline still occurs. In contrast, when both the (CdS)_34_:Ag MSCs and Ag NPs are crosslinked, the ECL intensity remained stable even after five consecutive scan cycles. The enhanced stability can be explained by the fact that the stability of the film contributes to the formation of a homogeneous electrode interface, reduces non-specific adsorption and degradation, and ensures the reproducibility of the ECL reaction after prolonged use. Such improved stability plays a crucial role in optimizing the performance of electrochemical luminescence systems, making them more effective for various applications in sensing and imaging.

### Stable MSC-based BPE-ECL platform for glucose detection

The integration of doping and crosslinking strategies has significantly improved the performance of BPE-ECL systems. This dual approach ensures that both the emitter, (CdS)_34_:Ag MSCs, and the catalyst, Ag NPs, remained firmly anchored to the electrode surface, mitigating material loss and enhancing reproducibility. Building on these advancements, we developed a BPE-ECL platform for highly sensitive glucose detection. This platform demonstrates remarkable sensitivity, stability, and practical applicability in real sample analysis, highlighting its potential for diverse bioanalytical applications.

**Fig. 4:**
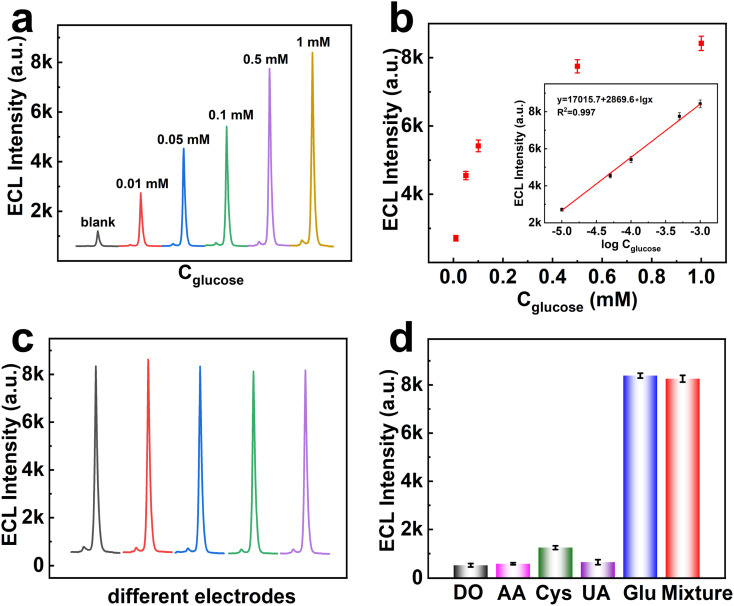
(a) ECL-time response signals of the biosensor for different concentrations of glucose (10 μM–1 mM). (b) Relationship between ECL (deduct background) and glucose concentration (inset: the logarithmic calibration plot for glucose detection). (c) ECL-time response signals for glucose detection by different electrode biosensors. (d) Selectivity of the electrochemical biosensor to glucose (0.5 mM) in comparison with dopamine (DO), ascorbic acid (AA), cysteine (Cys), uric acid (UA) and a mixture.

As shown in Fig. 4a, the ECL signals of the BPE-ECL system increase proportionally with rising glucose concentrations, demonstrating the biosensor's high efficacy for glucose detection. Fig. 4b further confirms a linear relationship (*R*^2^ = 0.997) between ECL intensity and glucose concentration over a range of 10 μM to 1 mM, achieving a detection limit (LOD) of 3.64 μM. A comparison of our approach with other reported methods in Table S2† revealed that our method exhibited markedly enhanced sensitivity for glucose detection. In this system, glucose oxidase (GOD) catalyzes the oxidation of glucose to gluconic acid, releasing hydrogen peroxide (H_2_O_2_) as a byproduct (Scheme 1a). Under voltage excitation, H_2_O_2_ decomposes into H_2_O and O_2_, accelerating electron transfer within the BPE-ECL system. This enhanced electron transfer amplifies the ECL intensity of (CdS)_34_ MSCs, enabling highly sensitive and reliable glucose detection.

The reproducibility and selectivity of ECL biosensors are essential metrics for evaluating their performance. High reproducibility ensures that the sensor's responses remain consistent over time and across different batches, a crucial requirement for routine clinical diagnostics and research applications. Fig. 4c presents the ECL stability of the BPE system in potential scans for 5 different electrodes. It was found that the biosensor displayed good ECL stability and reproducibility, and the calculated RSD was 1.25%, which made it applicable for glucose-related disease monitoring. Selectivity, on the other hand, determines the biosensor's ability to distinguish the target analyte from potential interfering substances in complex sample matrices. A selective biosensor minimizes cross-reactivity and false positives, thereby enhancing the accuracy of measurements. The selectivity of the BPE system for glucose detection in the presence of some biomolecules was tested. It was observed that the ECL signal response for glucose (150 μM) was significantly higher than that to other substances (0.5 mM) (Fig. 4d), which proved that our proposed biosensor showed high selectivity to glucose and could meet application requirements. Together, high reproducibility and selectivity not only enhance the credibility of the ECL biosensor but also expand its applicability in diverse fields, including medical diagnostics, environmental monitoring, and food safety testing.

The application of ECL biosensors for glucose detection in real samples, such as human serum, demonstrates their practical relevance in clinical diagnostics by a standard addition method. The samples were spiked with a standard glucose solution at concentrations of 0.01, 0.1, and 1 mM, respectively, to assess the recovery rate. The accuracy of the results was evaluated by relative standard deviation (RSD). The measured results are displayed in Table 1, and the recovery and RSD values were in the ranges of 92.21–105.96% and 4.2–6.3%, respectively, indicating that the ECL biosensor leverages its sensitivity and selectivity to provide accurate measurements despite the complex composition of biological fluids. This development highlights the versatility and effectiveness of the BPE-ECL system in addressing practical analytical challenges in the field of biosensing.

**Table 1:** Recovery results of glucose determination with the proposed BPE-ECL system in diluted serum samples

Sample	Add/mM	Recovery/%	RSD/%
1	0.01	99.35	5.7
2	0.1	92.21	4.2
3	1	105.96	6.3

## Conclusion

The implementation of doping and crosslinking strategies has resulted in significant enhancements in both the intensity and the stability of semiconductor nanoclusters. Doping precisely tunes the electronic properties of MSCs, leading to a marked increase in ECL intensity, while crosslinking reinforces the mechanical integrity of the nanocluster films, ensuring that the emitters and catalysts remain securely anchored to the electrode surface. These advancements contribute to the development of a highly reliable and robust biosensing platform. Based on these results, we developed a BPE-ECL biosensing platform tailored for the ultrasensitive detection of glucose. This platform not only demonstrated exceptional sensitivity and stability but also proved its practical applicability by successfully analyzing real samples, such as human serum, showcasing its potential for clinical applications. The successful realization of this biosensing platform underscores the transformative role of semiconductor nanoclusters in advancing analytical methodologies, paving the way for their broader use in bioanalysis and medical diagnostics.

## Author contributions

Y. W. conceived the concept of this work. Y. W. and J. G. co-wrote the paper. J. G. and T. Y. designed the experiments and analyzed the data. H. Z., Y. C., and J. L. performed optical and SEM measurements and photolithography. Y. Z., Y. W., and J. Z. supervised the project.

## Conflicts of interest

The authors declare no competing financial interests.

## Supplementary Material

SC-016-D4SC07800D-s001

## Data Availability

The data supporting this article have been included as part of the ESI.†
