# Supplementary material for: Photo-crosslinking of doped magic-sized nanoclusters for the construction of enhanced electrochemiluminescence biosensors
Source: Chem Sci. 2025 Jan 22;16(8):3671–9. doi: 10.1039/d4sc07800d (PMC11774253; doi:10.1039/d4sc07800d)
Supplement: SC-016-D4SC07800D-s001 [file SC-016-D4SC07800D-s001.pdf]

Supplementary Information (SI) for Chemical Science.
This journal is © The Royal Society of Chemistry 2025

Photo-Crosslinking of Doped Magic-size Nanocluster for the Construction of Enhanced Electrochemiluminescence Biosensors

Junjun Ge[#†], Tengyue Yin[#†], Haoyang Zhang[†], Yue Cao[††], Juan Liu[†††,††††], Jun-Jie Zhu[†], Yang Zhou[††]*, Yuanyuan Wang[†]*

[†] *State Key Laboratory of Coordination Chemistry, School of Chemistry and Chemical Engineering, Nanjing University, Nanjing 210093, China.*

†† *Key Laboratory for Organic Electronics & Information Displays and Institute of Advanced Materials, Nanjing University of Posts & Telecommunications (NJUPT), Nanjing 210023, PR China.*

†††*State Key Laboratory of Pollution Control and Resource Reuse , School of the Environment, Nanjing University, Nanjing 210023, China*

††††*School of Environmental & Chemical Engineering, Jiangsu University of Science and Technology, Zhenjiang 212100, China*

[#] The authors contribute equally to this work.

* To whom correspondence should be addressed.

E-mails: iamyangzhou@njupt.edu.cn, wangyy@nju.edu.cn

Experimental Section

Materials and Reagents All chemicals were commercially available and used as received without further purification n-octylamine (OTA, 99%) were purchased from Aladdin Co., Ltd. (Shanghai, China). Sulfur (99.999%) were obtained from Alfa Aesar (Shanghai, China). Tri-n-octylphosphine (TOP) were obtained from Strem Chemical Co., Ltd. (Xi′an, China). Butylamine (BTA, AR, ≥99.0%) and toluene (AR, ≥99.5%)were obtained from Sinopharm Chemical Reagent Co., Ltd.

Synthesis of $(CdS)_{34}$-OTA MSCs. The procedure for the synthesis of $(CdS)_{34}$-OTA was adapted from Hyeon group.[1] $CdCl_2$(octylamine)$_2$ complex was prepared by heating octylamine (10 mL) containing $CdCl_2$ (1.5 mmol) to 120 °C and aging at that temperature for 2 h under N_2. Sulfur-octylamine complex was prepared by dissolving elemental sulfur (4.5 mmol) in octylamine (5 mL) at room temperature. The resulting reddish-brown sulfur-octylamine complex solution was injected into $CdCl_2$(octylamine)$_2$ complex solution at room temperature and the color of the solution turned immediately to transparent light yellow. The reaction mixture was heated from room temperature to 40 °C. Finally, 1.5 mL of TOP was added to stop the reaction. The white precipitate was separated using a benchtop centrifuge (10000rpm, 30 s) at room temperature, and the colorless supernatant was discarded. The remaining white slush was re-dispersed into toluene or hexane to dilute 5 times.

Treating $(CdS)_{34}$-OTA with AgOTf. In a typical process, different volumes (200μL, 100μL, 66μL, 33μL, 11μL) of AgOTf solution (1mg/100μL in toluene) was added to

the purified $(CdS)_{34}$-OTA MSCs (1 mL), resulting in a small amount of brown-black precipitate and brown-yellow supernatant after 3 h. The supernatant was separated using a benchtop centrifuge (10000rpm, 30 s) at room temperature, and the precipitate was discarded. The supernatant was purified by precipitating with an equal volume of ethanol followed by centrifugation, and then re-dispersed into toluene.

Crosslinking and patterning of MSCs.

Synthesis of photosensitive molecule (3,3'-(perfluorobutane-1,4-diyl)bis(4,1-phenylene))bis(3-(trifluoromethyl)-3H-diazirine)). The synthesis of photosensitive molecule used reported protocols.[2] In a 250 mL flask, 13 mL of anhydrous DMSO, 5.5 g of 4'-bromo-2,2,2-trifluoroacetophenone, 195 μL of 1,1,2,2,3,3,4,4-octafluoro-1,4-diiodobutane, 12.71 g of copper powder, and 0.68 g of pyridine were mixed under nitrogen. The mixture was heated to 80 °C and stirred for 17 h. After being cooled to room temperature, 25 mL of water and 100 mL of ethyl acetate were added, followed by filtering. The filtrate was extracted with 50 mL of ethyl acetate for threetimes. The products in the organic phase were combined and washed with 50 mL of water and 50 mL of brine. The extract was then dried over anhydrous Na_2SO_4. After solvent removal by rotary evaporation and rinse with dichloromethane, a white solid (1,1'-((perfluorobutane-1,4-diyl)bis(4,1-phenylene))bis(2,2,2-trifluoroethan-1-one)) was obtained.

1.5 g of 1,1'-((perfluorobutane-1,4-diyl)bis(4,1-phenylene))bis(2,2,2-trifluoroethan-1-one), 1.14 g of hydroxylammonium chloride and 15 mL of pyridine were added in a 2-necked flask under nitrogen. The mixture was heated to 70 °C and stirred for 6 h. After the reaction, excess pyridine was removed by rotary evaporation. The product was then washed with 15 mL of water and 50 mL of ethyl acetate, followed by twice extraction with 25 mL of ethylacetate. Products in the organic phase were collected and washed by a sequence of 15 mL of water, 10 mL of HCl (twice), 10 mL of water (twice), and 15 mL of brine. Then, the extract was dried over anhydrous Na_2SO_4 and a white solid of 1,1'-((perfluorobutane-1,4-diyl)bis(4,1-phenylene))bis(2,2,2-trifluoroethan-1-one) dioxime was obtained.

1.25 g of 1,1'-((perfluorobutane-1,4-diyl)bis(4,1-phenylene))bis(2,2,2-trifluoroethan-1-one) dioxime and 28 mL of dichloromethane were mixed in a 100 mL flask and cooled to 0 °C in ice water. 127 μL of trimethylamine was added dropwise into the flask under stirring. During stirring, the white, turbid liquid turned into a yellow liquid. 1.37 g of toluenesulfonyl chloride and 5 mg of DMAP were then introduced. The mixture

was stirred for 17 h at room temperature and diluted with dichloromethane. The product in organic phase was washed by a sequence of 15 mL of water, 10 mL of NH_4Cl solution (twice), 10 mL of water (twice), and 15 mL of brine. The combined extract was dried over anhydrous Na_2SO_4, yielding a white solid of 1,1'-((perfluorobutane-1,4-diyl)bis(4,1-phenylene))bis(2,2,2-trifluoroethan-1-one) O,O-ditosyl dioxime.

1.3 g of 1,1'-((perfluorobutane-1,4-diyl)bis(4,1-phenylene))bis(2,2,2-trifluoroethan-1-one) O,O-ditosyl dioxime, 10 mL of dichloromethane and anhydrous tetrahydrofuran was mixed. The mixture was added dropwise into 50 mL of liquid ammonia at –78 °C. At the completion of the addition, the liquid ammonia bath was removed. The mixture was kept stirring for 14 h. The obtained turbid liquid was washed by 75 mL of ethyl acetate and 10 mL of water. The mixture was extracted by 25 mL of ethyl acetate. The organic phase was collected, followed by rinsing with 15 mL of water and 25 mL of brine. The extract was dried over anhydrous Na_2SO_4 and purified by using silica gel column chromatography. A white solid of 3,3'-((perfluorobutane-1,4-diyl)bis(4,1-phenylene))bis(3- (trifluoromethyl)diaziridine) was obtained by using petroleum ether (containing 5%–20% ethyl acetate) as the mobile phase.

Under nitrogen, 0.5 g of 3,3'-((perfluorobutane-1,4-diyl)bis(4,1-phenylene))bis(3-(trifluoromethyl)diaziridine) and 5 mL of dichloromethane were mixed in a 3-necked 100 mL flask in an ice water bath. 730 μL of triethylamine was added dropwise in the flask. Next, 0.47 g of iodine was added slowly under stirring. After 2 h, the mixture was diluted with 15 mL of dichloromethane and then washed by a sequence of 10 mL of water, 5 mL of $Na_2S_2O_3$ for twice, 10 mL of water for twice and 15 mL of brine. Then, the extract was dried over anhydrous Na_2SO_4, and purified by using silica gel column chromatography. Photosensitive molecule was obtained as a white solid and it has the following molecular structure:

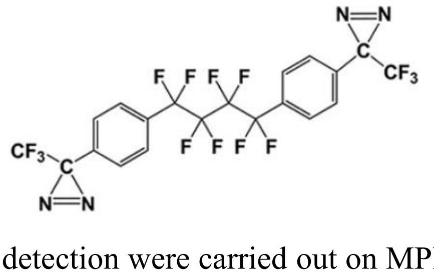

ECL Measurement. ECL detection were carried out on MPI-E ECL analyzer (Remex Electronic Instrument Lt. Co., Xi'an, China) with three-electrode system containing a Pt wire as the counter electrode, Ag/AgCl (saturated KCl) as the reference electrode, and a glassy carbon electrode as the working electrode. All MSCs were modified to the

surface of the glassy carbon electrode by drip coating then air-dry or vacuum dry. For $(CdS)_{34}$ MSCs, the ECL signals were recorded in the presence of 50 mM TPrA in 1:1 acetonitrile:toluene containing 0.1 M TBAP during the potential scan from 0 to 1.5 V at 1000 V of the photomultiplier tube (PMT).

ECL detection of glucose were carried out on MPI-E ECL analyzer with two-electrode system (BPE). The BPE bipolar electrode has a size of 3×6 cm, with ITO portions on both sides and in the center, which are used as the driver electrode and bipolar electrode, respectively. Cathodic treatment step: 10uL of n-octane solution of 60mg/mL Ag NCs was mixed with 2uL of n-octane solution of 30mg/mL photosensitive molecule (3,3′-(4,4′-(perfluorobutane-1,4-diyl)bis(4,1-phenylene))bis(3-(trifluoromethyl)-3H-diazirine)and spin-coated at 4000rpm for 40s, after which, it was irradiated under a UV lamp at 254nm for 4min under the shade of a mask, and then subsequently, it was developed with toluene to obtain the desired pattern of Ag NCs, after which it was cleaned under a plasma cleaner (Plasma) at 100 Pa for 30 s to remove the oleylamine groups on the surface of Ag NCs and make them hydrophilic. Anodic treatment step: 3 uL of 2.5 mg/mL $(CdS)_{34}$-Ag MSCs were drop-coated and dried at room temperature for 3h to form a film. The ECL signals were recorded in the presence of 50 mM TPrA in 1:1 acetonitrile:toluene containing 0.1 M TBAP during the potential scan from -8 to 8 V at 900 V of the PMT. Different concentrations of the target glucose were dissolved in PBS solution with sufficient amount of glucose oxidase (0.5 mg) and dropped in the cathodic detection bath.

Apart from this, we combined CHI760E electrochemical workstation and 3D fluorescence spectrometer (FL-3) to measure ECL spectra. The platinum sheet electrode was used as the working electrode, and the constant potential was 1.5 V or 0.9 V.

Raman Measurement. Laser confocal Raman spectra measurements were carried out using a Renishaw inVia-Reflex Raman system at room temperature. Excitation wavelengths of 488 nm (~20 mW) from a Ar-ion laser were selectively used to excite the $(CdS)_{34}$ MSCs.

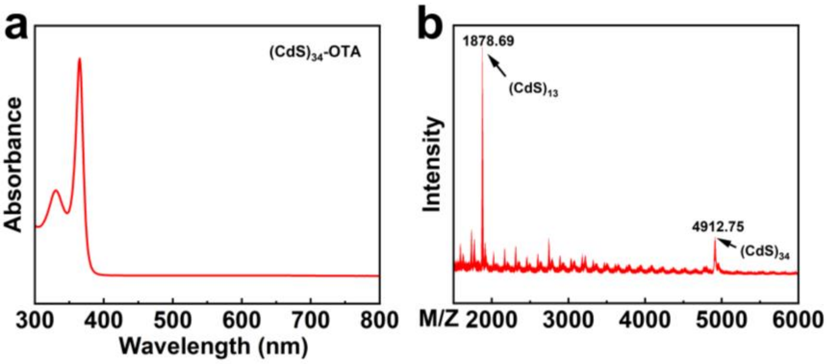

Figure S1. (a) Absorption spectrum and (b) MALDI of $(CdS)_{34}$ MSCs.

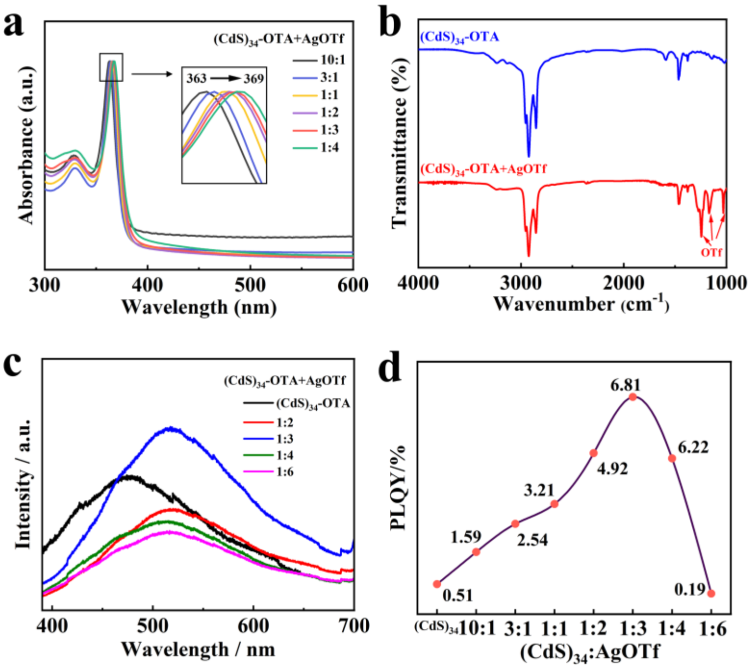

Figure S2. (a) Absorption (c) PL spectra and (d) PLQY of $(CdS)_{34}$:Ag MSCs with different feeding ratios. (b) IR spectra of $(CdS)_{34}$ and $(CdS)_{34}$:Ag MSCs.

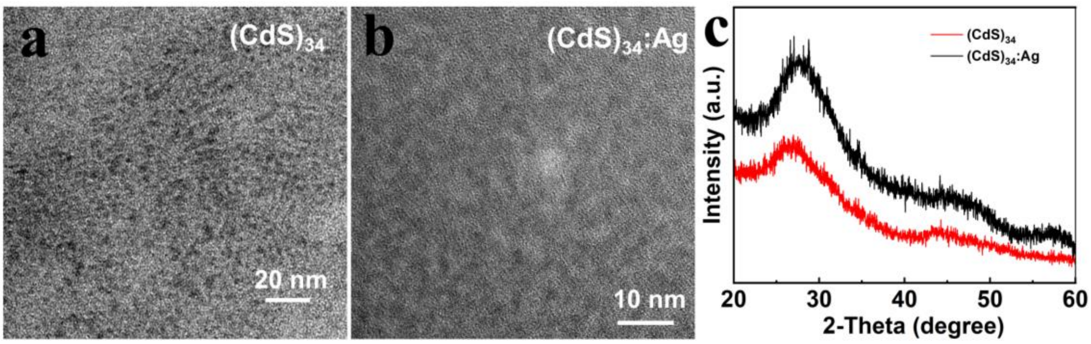

Figure S3 TEM images of (a) $(CdS)_{34}$ MSCs and (b) $(CdS)_{34}$:Ag MSCs.(c) XRD pattern of $(CdS)_{34}$ and $(CdS)_{34}$:Ag MSCs.

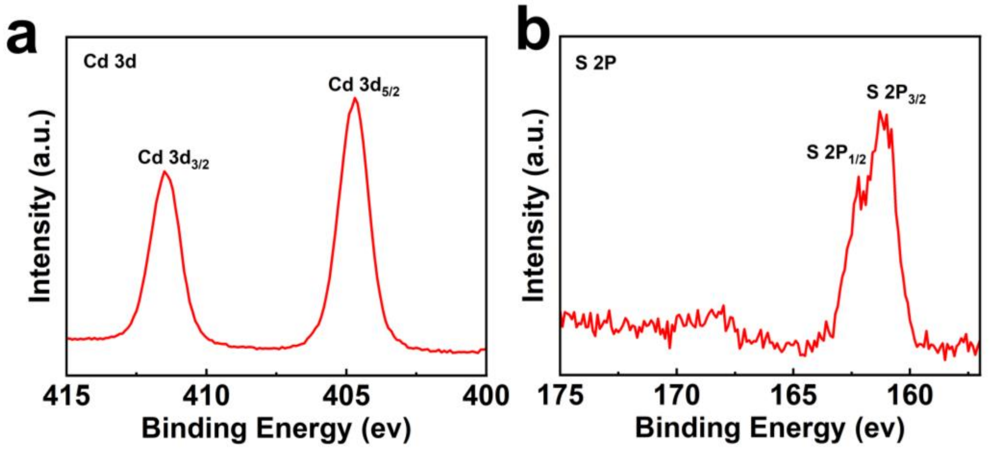

Figure S4 High-resolution X-Ray photoelectron spectra of $(CdS)_{34}$:Ag MSCs in the ranges of Ag3d electron binding energy.

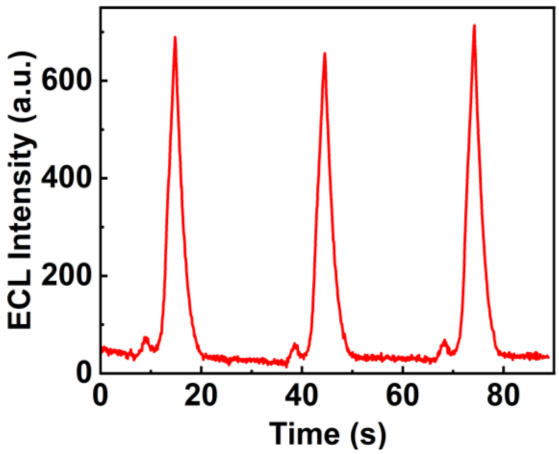

Figure S5. ECL-Time curve of $(CdS)_{34}$ MSCs

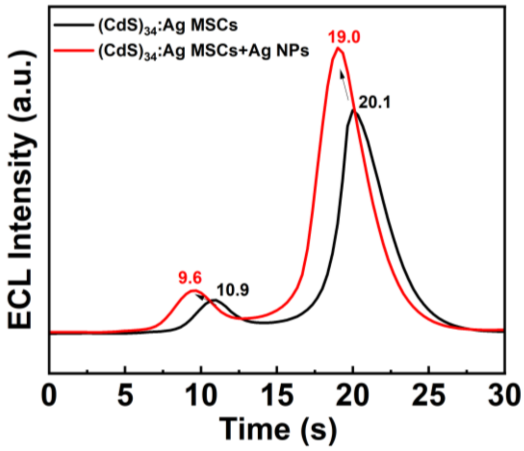

Figure S6 ECL-Time curves of $(CdS)_{34}$:Ag MSCs before and after Ag NPs catalysis

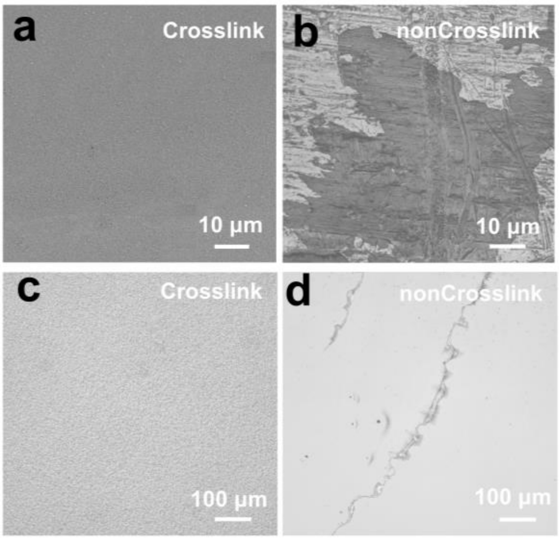

Figure S7 SEM image of (a) $(CdS)_{34}$:Ag MSCs with crosslink; (b) $(CdS)_{34}$:Ag MSCs without crosslink; (c) AgNPs with crosslink; (d) AgNPs without crosslink.

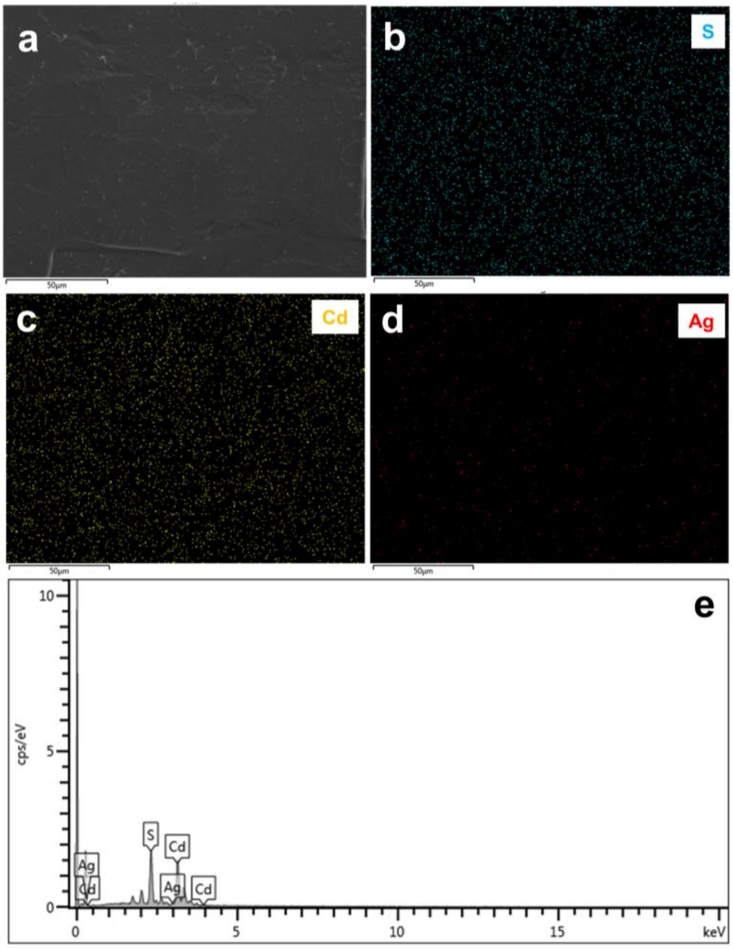

Figure S8 (a)SEM image of $(CdS)_{34}$:Ag MSCs with crosslink; Distribution of elements corresponding to (a): (b) S, (c) Cd, (d) Ag; (e) corresponding EDS energy spectra of $(CdS)_{34}$:Ag MSCs with crosslink.

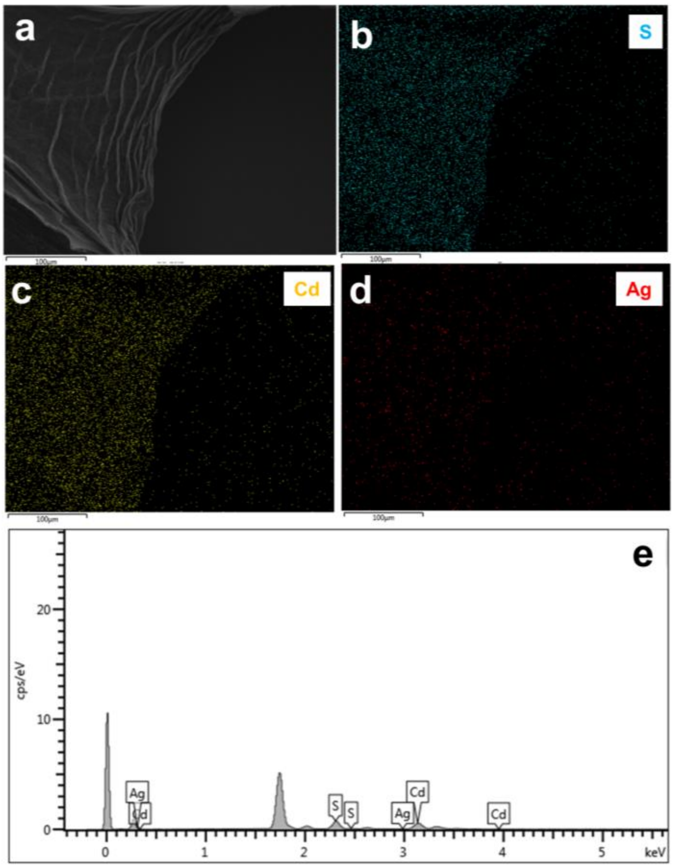

Figure S9 (a)SEM image of $(CdS)_{34}$:Ag MSCs without crosslink; Distribution of elements corresponding to (a): (b) S, (c) Cd, (d) Ag; (e) corresponding EDS energy spectra of $(CdS)_{34}$:Ag MSCs without crosslink.

Table S1. ICP-OES Data for $(CdS)_{34}$ Corresponding to Doping

	S	Cd	Ag	Cd:S:Ag
$(CdS)_{34}$	0.556	2.03	-	0.96:1
1:1	3.428	13.01	1.356	1.07:1:0.12
1:3	12.73	48.49	5.925	1.07:1:0.138

Table S2. Comparison of other assays using nanoclusters (NCs) as emitters with this experiment

Method and ref.	emitter	linear	LOD

ECL[3]	Au NCs	50 μM–3.0 mM	20 μM
ECL[4]	Au_{25} NCs	0.004 -90 ng/mL	0.001 ng/mL
fluorescent ratiometric[5]	CAT-Au NCs	5 μM-0.5 mM	3 μM
colorimetric[6]	Pt-LNT NCs	5 μM-1 mM	1.79 μM
absorbance[7]	Cu NCs	10 μM-5 mM	5.5 μM
electrochemical[8]	Co_3O_4 NCs	88 μM-7.0 mM	26 μM
fluorescence[9]	AuNCs@ZIF-8	0-1000 μM and 1-20 mM	4.7 μM
electrochemical[10]	DFs/AgNCs	50 μM-1 mM	20 μM
ECL (This work)	$(CdS)_{34}$:Ag MSCs	10 μM-1 mM	3.64 μM

1. Son, J. S.; Park, K.; Kwon, S. G.; Yang, J.; Choi, M. K.; Kim, J.; Yu, J. H.; Joo, J.; Hyeon, T. Dimension-controlled synthesis of CdS nanocrystals: from 0D quantum dots to 2D nanoplates. *Small* **2012,** 8, (15), 2394-402.
2. Liu, D.; Weng, K.; Zhao, H.; Wang, S.; Qiu, H.; Luo, X.; Lu, S.; Duan, L.; Bai, S.; Zhang, H.; Li, J. Nondestructive Direct Optical Patterning of Perovskite Nanocrystals with Carbene-Based Ligand Cross-Linkers. *ACS Nano* **2024,** 18, (9), 6896-6907.
3. Han, S.; Gao, Y.; Li, L.; Lu, B.; Zou, Y.; Zhang, L.; Zhang, J. Synergistic Enhancement Effects of Carbon Quantum Dots and Au Nanoclusters for Cathodic ECL and Non-enzyme Detections of Glucose. *Electroanalysis* **2020,** 32, (6), 1155-1159.
4. Chen, S.; Fan, Y.; Zhang, C.; He, Y.; Wei, S. Quenched solid-state electrochemiluminescence of gold nanoclusters and the application in the ultrasensitive detection of concanavalin A. *Electrochimica Acta* **2017,** 228, 195-202.
5. Meng, F.; Yin, H.; Li, Y.; Zheng, S.; Gan, F.; Ye, G. One-step synthesis of enzyme-stabilized gold nanoclusters for fluorescent ratiometric detection of hydrogen peroxide, glucose and uric acid. *Microchemical Journal* **2018,** 141, 431-437.
6. Dong, L.; Li, R.; Wang, L.; Lan, X.; Sun, H.; Zhao, Y.; Wang, L. Green synthesis of platinum nanoclusters using lentinan for sensitively colorimetric detection of glucose. *International Journal of Biological Macromolecules* **2021,** 172, 289-298.
7. Han, A.; Zhao, Y.; Wu, J.; Guo, J.; Xv, J. Self-assembled copper nanoclusters used to mimic peroxidase for glucose detection. *RSC Advances* **2024,** 14, (5), 3261-3266.
8. Wang, L.; Zhang, Y.; Xie, Y.; Yu, J.; Yang, H.; Miao, L.; Song, Y. Three-dimensional macroporous carbon/hierarchical Co3O4 nanoclusters for nonenzymatic electrochemical glucose sensor. *Applied Surface Science* **2017,** 402, 47-52.
9. Sun, Y.; Shu, T.; Ma, J.; Dai, Q.; Peng, P.; Zhou, Z.; Zhou, X.; Su, L.; Zhang, X. Rational Design

of ZIF-8 for Constructing Luminescent Biosensors with Glucose Oxidase and AIE-Type Gold Nanoclusters. *Analytical Chemistry* **2022,** 94, (7), 3408-3417.

10. Qiao, Z.; Yan, Y.; Bi, S. Three-dimensional DNA structures in situ decorated with metal nanoclusters for dual-mode biosensing of glucose. *Sensors and Actuators B: Chemical* **2022,** 352, 131073.
